# Dynamic scale fusion and cascaded prototype refinement for few-shot sports image classification

**DOI:** 10.3389/fpsyg.2026.1974654

**Published:** 2026-10-29

**Authors:** Dongfang Gao, Yuyan Ruan, Chun Chang, Wenzhu Yao

**Affiliations:** 1 Department of Basic and Public Courses, Jiaxing Vocational and Technical College, Jiaxing, Zhejiang, China; 2 School of Fashion and Art Design, Jiaxing Vocational and Technical College, Jiaxing, Zhejiang, China; 3 School of Medical Humanities, Anhui Medical University, Hefei, Anhui, China

**Keywords:** attention mechanism, deep learning, feature learning, few-shot learning, sports image recognition

## Abstract

**Inroduction:**

Sports image classification plays a crucial role in intelligent sports analytics by supporting athlete behavior analysis, training feedback, exercise participation monitoring, and data-driven sports behavior studies. Although deep learning methods have advanced sports image recognition by learning hierarchical visual patterns from image data, they usually require abundant annotations, which limits their applicability in few-shot scenarios. In addition, sports images often show large-scale variations, severe background distraction, and fine-grained inter-class similarities, further degrading recognition performance under scarce supervision.

**Methods:**

To address these challenges, this study proposes a dynamic scale fusion and cascaded prototype refinement network (DSCPNet) for few-shot sports image classification. First, Dynamic Scale Fusion Attention (DSFA) is introduced to dynamically select and fuse discriminative contextual cues under varying receptive fields, thereby enhancing scale-adaptive feature representation from scarce support samples. Second, Cascaded Foreground-Scene Interaction (CFSI) is designed to suppress background interference through cascaded interaction modulation, strengthening target-focused embeddings and reducing intra-class variance. Finally, Cross-Prototype Feature Refinement (CPFR) is developed to calibrate support prototypes and query representations in a cross-prototype manner, producing more discriminative class prototypes and improving the robustness of similarity estimation for fine-grained recognition.

**Results:**

Experiments on two sports-oriented few-shot benchmark datasets show that DSCPNet consistently achieves stronger recognition performance than competing methods in both 1-shot and 5-shot scenarios.

**Discussion:**

These results indicate that DSCPNet can improve few-shot sports image recognition under scale variation, background distraction, and fine-grained inter-class ambiguity. The proposed framework provides an effective solution for sports image classification when labeled samples are limited.

## 1 Introduction

Sports image classification plays a crucial role in sports understanding and analytics, since accurate recognition supports downstream applications such as event indexing, tactical analysis, broadcast production, and intelligent retrieval in large-scale multimedia platforms. Beyond multimedia management, reliable classification also facilitates automatic highlight generation, fine-grained statistical reporting, and cross-domain knowledge transfer for sports-related decision making, which reduces manual effort in large-scale content processing and improves the efficiency of sports information services. Meanwhile, sports imagery poses distinct challenges for visual recognition. Real-world sports images often exhibit large intra-class variation caused by diverse venues, uniforms, camera viewpoints, and illumination conditions, while inter-class similarity is common among sports that share similar objects or layouts. In addition, fast motion, partial occlusion, and frequent background clutter introduce distracting cues that can mislead feature learning, which make sports image classification a non-trivial task, especially when robust generalization is required across different events and data collection conditions. From the perspective of sport psychology-related applications, reliable sports image recognition can provide basic visual evidence for analyzing athlete behavior, training participation, exercise habits, and sport-scene interactions. For example, recognizing sports categories and action-related visual patterns from limited labeled samples may support downstream studies on athlete movement characteristics, training feedback, participation monitoring, and data-driven sport behavior analysis. Therefore, improving few-shot sports image classification is relevant not only to computer vision but also to intelligent sports analysis scenarios that provide visual information for sport psychology research and applications. Early recognition systems commonly followed hand-engineered pipelines, in which visual descriptors such as color, texture, shape, and local key points were first computed and then classified using models such as SVM, KNN, or decision trees (Song and Hagras, 2016). However, these feature-engineering-based approaches often exhibit limited robustness when facing large intra-class variation, viewpoint and illumination changes, occlusion, and background clutter in real sports imagery, which leads to performance degradation and weak generalization under complex environments.

Driven by the development of deep learning, CNN-based and Transformer-based architectures have greatly improved sports image classification through direct learning of hierarchical representations from raw data, thus lowering reliance on handcrafted features and enhancing discriminative performance. For example, Li Q. et al. (2024) developed a sports image recognition model based on squeeze and excitation block, which combines attention and residual mechanism to extract more features. Russo et al. (2018) developed a CNN model with recurrent neural networks for sports classification, which utilizes frames of training video as input to enhance the model performance. Zhang et al. (2023) developed a transformer-based model for football scenes recognition, which employs self-attention to promote the feature of objects with variable contextual information. Zhou et al. (2024) proposed a recognition model with graph architecture, which employs swarm optimization to obtain a model with high accuracy. Srilakshmi and Joe (2023) developed a sports classification model based on U-Net, which incorporates the residual attention to obtain more details. However, these methods typically depend on large-scale labeled datasets, while many sports categories remain data-scarce due to the high cost of annotation, long-tail class distributions, and the rarity of certain events or scenes. This limitation motivates the adoption of few-shot learning, which aims to recognize novel categories with only a few labeled examples and thus provides a practical paradigm for image understanding under limited supervision. Cao et al. (2025) designed an unsupervised few-shot framework for novel-class recognition, which generates augmented matching tasks and uses two coordinated encoders to learn transferable feature representations without relying on labeled base-class data. Wu et al. (2025) developed a few-shot method for human intention recognition in robot collaboration by adapting vision-language models. Liu et al. (2024) proposed a few-shot named entity recognition method that uses repulsion loss and evidential k-nearest neighbor inference to better exploit information from surrounding samples. Liao et al. (2025) introduced a radar signal recognition approach for limited-sample conditions, where prototype representations are refined by weighting sample reliability and adjusting decision boundaries for unknown classes. Shi et al. (2025) developed a limited-sample network attack detector by highlighting discriminative traffic features and incorporating unlabeled query samples during inference. Li X. et al. (2025) developed a limited-label hyperspectral classification method that transfers graph-based teacher knowledge to student networks and strengthens feature learning through graph augmentation and ensemble modeling.

Despite these advances, few-shot sports image classification still requires further investigation because sports images often contain scarce annotations, complex backgrounds, and fine-grained category differences. Deep learning-based sports scene recognition aims to categorize sports images into predefined scene classes by learning discriminative representations that jointly capture athlete-centered cues, scene layout, and high-level semantic context, which enables robust recognition under large intra-class variation, viewpoint changes, and complex background clutter. For example, Liu et al. (2026) proposed a data augmentation strategy for sports images, which aligns feature representations across different sports scenes to improve recognition accuracy. Gao and Katagishi (2016) developed a pyramid-based framework for sports image recognition, which enhances spatial information to jointly capture global and local features. Hsu et al. (2019) proposed a classification model for wearable sports analysis, which uses CNNs to process motion signals with feature normalization and enhancement. Hua and Han (2024) developed a sports image classification method based on a large language model, which generates frequency-domain representations to strengthen model generalization. Mizher et al. (2025) developed a CNN-based sports image recognition model, which is designed to suppress the adverse impact of background noise. Nevertheless, most sports recognition methods still rely on sufficient labeled samples, which limits their direct applicability to newly emerging or data-scarce sports categories. Beyond sports-specific recognition studies, recent action recognition methods have also explored vision-language and semantic reasoning paradigms. Wang M. et al. (2025) developed ActionCLIP, which adapts language-image pre-trained models to video action recognition by reformulating action recognition as a video-text matching problem and exploiting label semantics to improve transferability. Chen et al. (2026) proposed Vision-Semantics-Label (VSL), a two-step action recognition paradigm based on large language models, which maps visual information to semantic descriptions and then to action labels to strengthen semantic understanding in action recognition.

Building on this general few-shot learning background, recent studies have further explored feature enhancement, prototype construction, and contextual modeling to improve recognition under limited supervision and complex visual conditions. For instance, Liu et al. (2023) developed a few-shot model for image classification, which employs multi-resolution cropping and self-supervised learning to extract features with large receptive fields. Yan et al. (2025a) introduced a frequency-aware few-shot detection method that enhances visual representations and alleviates data imbalance effects. Zhao et al. (2026) developed a self-supervised few-shot framework, which leverages multi-branch features to construct representative class prototypes. Li Y. et al. (2025) introduced a few-shot PCB defect detection method to enhance defect-related features and support-query information fusion. Yan et al. (2025b) developed a cross-domain few-shot detection approach that strengthens target-domain adaptation by refining supervision signals and reducing uncertainty during transfer. Since few-shot classification relies heavily on the quality of feature representations, attention mechanisms have also been widely used to enhance discriminative cues and reduce irrelevant responses.

Specifically, attention mechanisms are designed to adaptively emphasize informative cues and suppress redundant disturbances, which enables neural networks to selectively allocate computational focus to task-relevant regions or channels. This paradigm has been widely integrated into CNN- and Transformer-based architectures, where it consistently improves feature representation and robustness across diverse vision tasks. Zhao and Yu (2025) developed a detection method, which introduces a boundary-aware attention module to enhance fine-grained feature extraction. Kong et al. (2026) designed a Transformer-based attention block for industrial fault detection, which strengthens the modeling of long-range dependencies. Wang Y. et al. (2025) designed a temporal attention method for limited-sample time-series analysis, which performs attention-guided augmentation to improve generalization. Xu et al. (2025) proposed a power usage prediction model, which employs attention to refine feature weighting and produce more reliable estimations. Shan et al. (2026) developed a remote sensing detection network, which incorporates an entropy-guided attention block to encourage the model to focus on uncertain regions. Taken together, these studies provide useful foundations for sports image recognition, few-shot learning, and attention-based feature enhancement, but several challenges remain when these techniques are applied to few-shot sports image classification.

Although few-shot learning has achieved encouraging results in visual recognition, applying it to sports images still involves several unresolved challenges: (1) In real sports images, key discriminative cues may appear at significantly different spatial scales (e.g., a small ball versus full-body posture), while a fixed receptive field fails to capture the most informative context, leading to unstable feature representations under few-shot supervision. (2) Sports images often include complex stadium backgrounds, audience regions, advertisements, and multiple irrelevant players, which introduce strong interference and dilute foreground semantics, making few-shot feature learning highly sensitive to noise and resulting in large intra-class variance. (3) Many sports actions or categories share highly similar visual patterns (e.g., running vs. dribbling, serving vs. smashing), and the prototypes computed from scarce support examples are often biased or incomplete, which degrades similarity measurement and causes severe inter-class confusion.

To solve the problems above, this study proposes a dynamic scale fusion and cascaded prototype refinement network (DSCPNet) for few-shot sports image classification. The main contributions of this study can be summarized as follows: (1) To address the scale diversity of sports objects and actions, Dynamic Scale Fusion Attention (DSFA) is proposed to dynamically select and fuse discriminative contextual cues under varying receptive fields, which enables more robust representation learning from scarce support samples. (2) To suppress severe background distraction in sports scenes, Cascaded Foreground–Scene Interaction (CFSI) is introduced to model foreground-aware and scene-aware feature responses through cascaded interaction modulation. These branches are termed foreground-aware and scene-aware according to their intended functional roles, which are used to enhance target-related responses and capture contextual interference, respectively. (3) To improve prototype matching reliability in fine-grained sports recognition, Cross-Prototype Feature Refinement (CPFR) is developed to refine support and query representations in a cross-prototype manner, which yields more discriminative class prototypes and robust similarity estimation.

The rest of the manuscript is organized in the following manner. Section 2 illustrate the proposed DSCPNet for few-shot sports image classification. Section 3 presents comprehensive experiments on two representative sports image datasets to assess the performance of DSCPNet against state-of-the-art methods. Section 4 presents the limitations and Discussion on DSCPNet. Section 5 makes a conclusion.

## 2 Dynamic scale fusion and cascaded prototype refinement network

### 2.1 Network structure of DSCPNet

In this paper, a dynamic scale fusion and cascaded prototype refinement network (DSCPNet) is proposed for few-shot sports image classification under a meta-learning paradigm. Two datasets are considered, including a base dataset 𝐷base for model training and a novel dataset 𝐷novel for evaluation, where 𝐷base∩𝐷novel = ∅. During meta-training, episodic tasks are sampled from 𝐷base to learn transferable representations. During meta-testing, unseen categories from 𝐷novel are organized into a support set 𝐷sup and a query set 𝐷que under an N-way K-shot setting, where N denotes the number of novel classes and K denotes the number of labeled examples per class in 𝐷sup. During meta-testing, samples from 𝐷novel are used only to construct support and query episodes for evaluation. They are not used to update model parameters or tune hyperparameters. The details are as follows: Given an episode, the support set and query set are first constructed. Each image is fed into a ResNet-12 backbone to extract convolutional feature maps. Subsequently, the extracted features are enhanced by Dynamic Scale Fusion Attention, which improves multi-scale context perception and strengthens discriminative cues. Then, Cascaded Foreground Scene Interaction is introduced to refine the enhanced representations by suppressing irrelevant background interference and reinforcing foreground-related responses. After feature refinement, Cross-Prototype Feature Refinement is applied to generate calibrated class prototypes and refined query embeddings, which alleviates prototype bias caused by scarce support samples. Finally, cosine similarity is computed between the query embedding and each refined prototype to obtain classification scores, and the query label is predicted according to the highest similarity response. The architecture of DSCPNet is described at two levels: Figure 1 presents the overall workflow, while Figures 2–4 provide the detailed structures of DSFA, CFSI, and CPFR, respectively.

**Figure 1:**
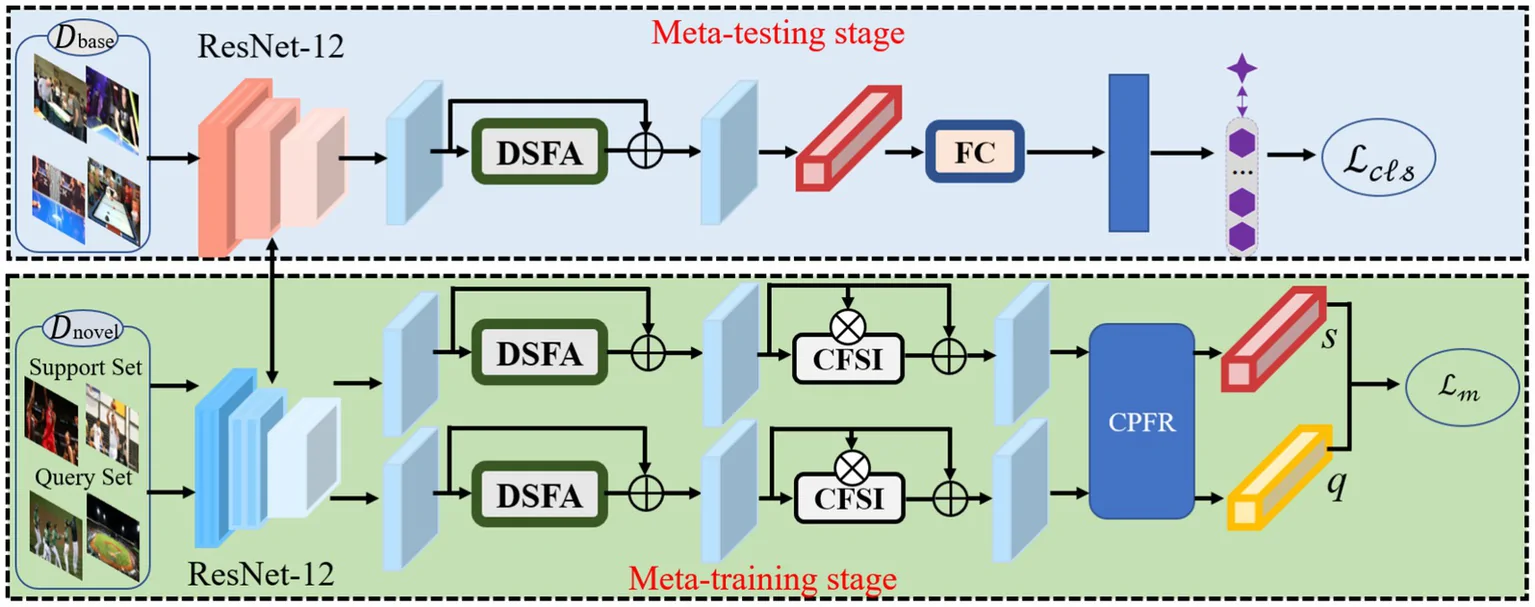
Dynamic scale fusion and cascaded prototype refinement network.

**Figure 2:**
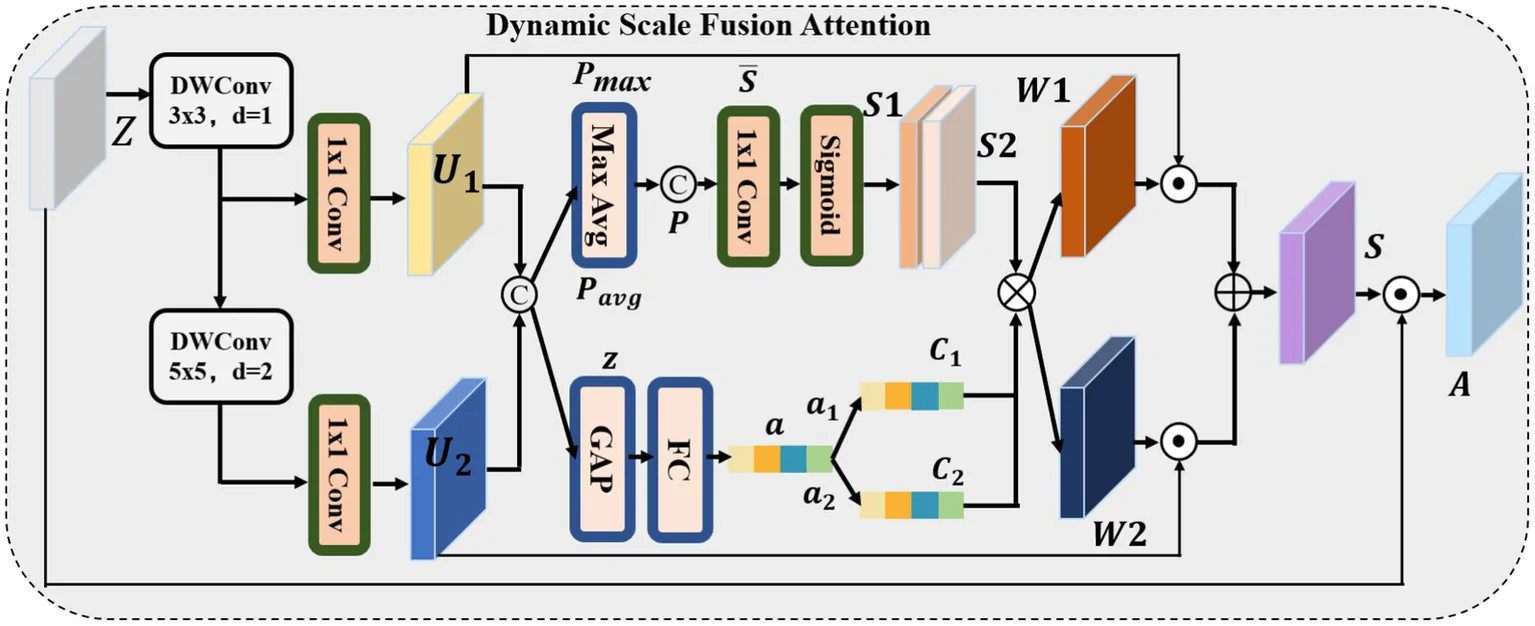
Structure of DSFA.

**Figure 3:**
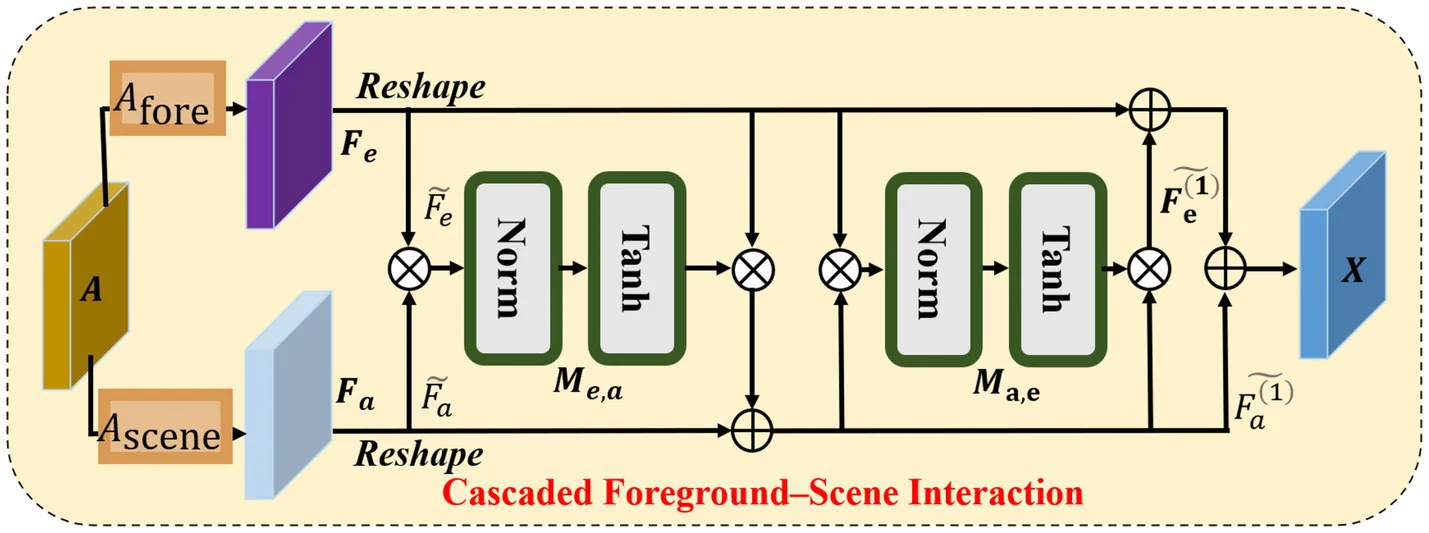
Structure of CFSI.

**Figure 4:**
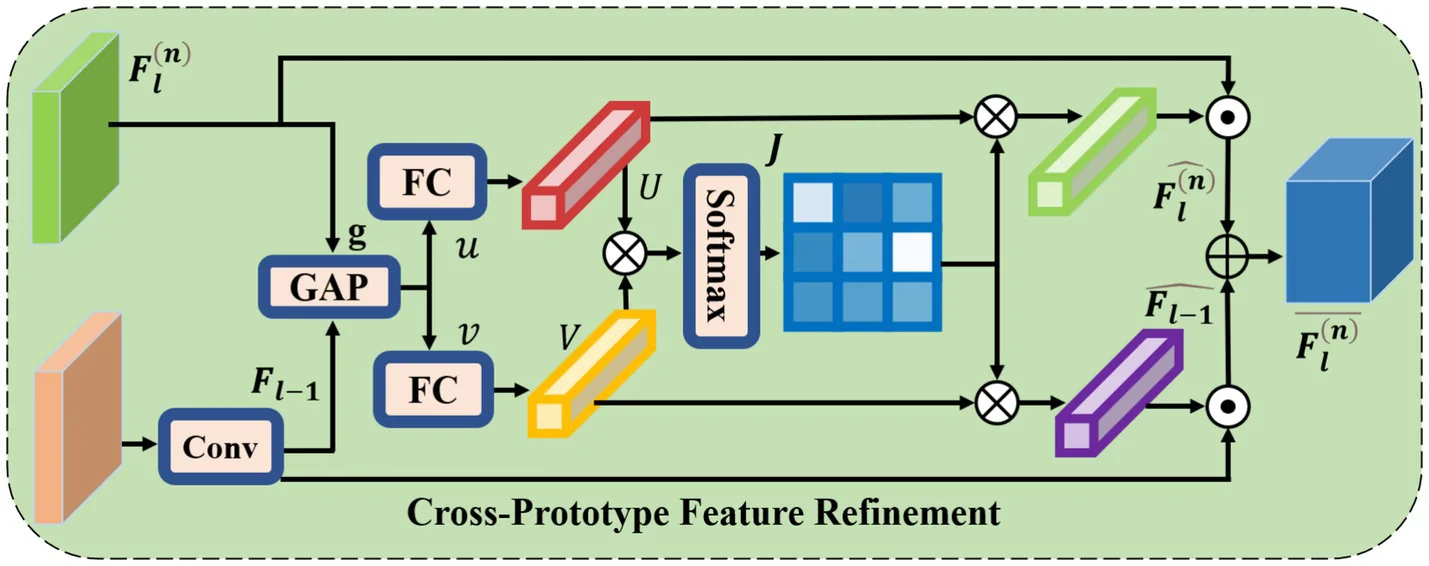
Structure of CPFR.

### 2.2 Dynamic scale fusion attention

Sports images often contain discriminative visual cues distributed at diverse spatial scales, such as small balls, rackets, and subtle limb motions, while the global body posture and scene context may dominate large receptive fields. A fixed receptive field is insufficient to capture the most informative context for different sports categories, which leads to unstable feature representations under few-shot supervision. To address this limitation, Dynamic Scale Fusion Attention (DSFA) is proposed to dynamically select and fuse multi-scale contextual features, which enhances scale-adaptive representation learning. The structure of DSFP is shown in Figure 2. Given an image 𝐼, the feature map Z extracted from ResNet12 is defined in Equation 1 as follows:

$$Z=f_{\theta }(I),Z\in R^{H\times W\times C}$$ (1)

Then, two scale-aware features are constructed with different receptive fields by two depth-wise convolution branches followed by 1 × 1 convolutions, as defined in Equation 2:

$$\begin{array}{l} U_{1}=\phi_{1\times 1}^{\left(1\right)}({\text{DWConv}}_{3\times 3,d=1}(Z)),U_{2}=\phi_{1\times 1}^{\left(2\right)}\\ \;\;\;\;\;\;\;\;\;\;\;({\text{DWConv}}_{5\times 5,d=2}(Z)),U_{1},U_{2}\in R^{H\times W\times C} \end{array}$$ (2)

Next, the two features are concatenated as defined in Equation 3:

$$U=[U_{1};U_{2}],U\in R^{H\times W\times 2C}$$ (3)

Scale-specific spatial selection masks are obtained by applying channel-wise max pooling and average pooling on *U*, which are then concatenated and mapped by a convolution layer followed by a sigmoid function, as defined in Equations 4–6:

$$P_{\max}={\text{MaxPool}}_{c}(U);P_{\mathrm{avg}}={\text{AvgPool}}_{c}(U)$$ (4)

$$P=[P_{\max};P_{\mathrm{avg}}],P\in R^{H\times W\times 2};\bar{S}=\text{Conv}(P),\bar{S}\in R^{H\times W\times 2}$$ (5)

$$[S_{1},S_{2}]=\sigma (\bar{S}),S_{1},S_{2}\in R^{H\times W\times 1}$$ (6)

Channel-wise scale coefficients are computed by applying global average pooling and a fully-connected layer on *U*, followed by a two-branch softmax normalization to generate C1 and C2, as defined in Equations 7, 8:

$$z=\mathrm{GAP}(U);a=\mathrm{FC}(z);[a_{1},a_{2}]=\text{split}(a)$$ (7)

$$C_{1}=\frac{\exp(a_{1})}{\exp(a_{1})+\exp(a_{2})};C_{2}=\frac{\exp(a_{2})}{\exp(a_{1})+\exp(a_{2})}$$ (8)

Then, spatial-channel fusion weights are formed as defined in Equation 9:

$$W_{1}=S_{1}⊗C_{1},W_{2}=S_{2}⊗C_{2};W_{1},W_{2}\in R^{H\times W\times C}$$ (9)

The fused representation is computed as defined in Equation 10:

$$S=W_{1}⊙U_{1}+W_{2}⊙U_{2},S\in R^{H\times W\times C}$$ (10)

Finally, feature re-calibration is applied to obtain the enhanced output, as defined in Equation 11:

$$A=Z⊙S,A\in R^{H\times W\times C}$$ (11)

where 𝛾 is a learnable coefficient.

### 2.3 Cascaded foreground–scene interaction

Sports images are usually captured in unconstrained environments, where background regions occupy large areas and contain strong texture patterns, which easily dominate feature activations and dilute discriminative foreground cues. Such background interference is particularly harmful under few-shot supervision, where limited support samples cannot provide sufficient statistical coverage.

To mitigate this issue, Cascaded Foreground–Scene Interaction (CFSI) is proposed to model complementary target-related and context-related feature responses through cascaded interaction modulation. In this study, the terms foreground-aware and scene-aware are used to describe the functional roles of two learned projection branches, rather than explicitly supervised semantic foreground masks or scene segmentation maps. The work flow of CFSI is displayed in Figure 3 and the details are as follows: After obtaining the DSFA-enhanced feature *A* $\in R^{C\times H\times W}$ , two independent projection branches are used to generate the foreground-aware feature $F_{e}\in R^{C\times H\times W}$ and the scene-aware feature $F_{a}\in R^{C\times H\times W}$ . Specifically, $A_{\text{fore}}(.)$ and $A_{\text{scene}}(.)$ denote two independent projection functions with non-shared parameters. Each projection function consists of a 1×1 convolution, batch normalization, and ReLU activation, and keeps the same spatial resolution and channel dimension as A . The two features are formulated in Equation 12 as follows:

$$\begin{array}{l} F_{e}=A_{\text{fore}}(A)=\delta (\mathrm{BN}({\text{Conv}}_{1\times 1}^{\text{fore}}(A))),\\ F_{a}=A_{\text{scene}}(A)=\delta (\mathrm{BN}({\text{Conv}}_{1\times 1}^{\text{scene}}(A))) \end{array}$$ (12)

where ${\text{Conv}}_{1\times 1}^{\text{fore}}$ and ${\text{Conv}}_{1\times 1}^{\text{scene}}$ denote two independent 1×1 convolutional layers with non-shared parameters, BN denotes batch normalization, and δ(.) denotes the ReLU activation function. It should be noted that $F_{e}$ and $F_{a}$ are not generated from explicit foreground annotations, segmentation masks, or object detectors. Instead, they are learned feature responses optimized under the few-shot classification objective. The branch $A_{\text{fore}}(.)$ is designed to emphasize target-related discriminative cues, while $A_{\text{scene}}(.)$ captures complementary scene and background-related contextual responses. Therefore, the terms foreground-aware and scene-aware indicate the functional roles of the two branches in the CFSI module, rather than pixel-level semantic supervision. Next, the features above are reshaped into matrices as defined in Equation 13:

$$\tilde{F_{e}}=\text{reshape}(F_{e})\in R^{C\times \mathrm{HW}},\tilde{F_{a}}=\text{reshape}(F_{a})\in R^{C\times \mathrm{HW}}$$ (13)

A foreground-to-scene interaction matrix is constructed by transpose multiplication followed by normalization and a tanh(·) nonlinearity, as defined in Equation 14:

$$M_{e,a}=\tanh(\text{Norm}(\tilde{F_{e}^{⊤}}⊗\tilde{F_{a}})),M_{e,a}\in R^{\mathrm{HW}\times \mathrm{HW}}$$ (14)

Then, foreground-aware cues are projected into the scene space to modulate the scene-aware feature, as shown in Equation 15:

$$\tilde{F_{a}^{\left(1\right)}}=\tilde{F_{e}}⊗M_{e,a}+\tilde{F_{a}},\tilde{F_{a}^{\left(1\right)}}\in R^{C\times \mathrm{HW}}$$ (15)

Subsequently, a symmetric scene-to-foreground interaction matrix is computed in the same operation, as defined in Equation 16:

$$M_{a,e}=\tanh(\text{Norm}({\left(\tilde{F_{a}^{\left(1\right)}}\right)}^{⊤}⊗\tilde{F_{e}})),M_{a,e}\in R^{\mathrm{HW}\times \mathrm{HW}}$$ (16)

The modulated scene-aware cues are further projected back to refine the foreground-aware feature, as shown in Equation 17:

$$\tilde{F_{e}^{\left(1\right)}}=\tilde{F_{a}^{\left(1\right)}}⊗M_{a,e}+\tilde{F_{e}},\tilde{F_{e}^{\left(1\right)}}\in R^{C\times \mathrm{HW}}$$ (17)

Finally, both refined matrices are reshaped back to feature maps and aggregated with a residual connection to produce the refined output, as defined in Equation 18:

$$X=A+{\text{reshape}}^{-1}(\tilde{F_{e}^{\left(1\right)}})+{\text{reshape}}^{-1}(\tilde{F_{a}^{\left(1\right)}}),X\in R^{C\times H\times W}$$ (18)

### 2.4 Cross-prototype feature refinement

In metric-based few-shot classification, class prototypes are typically obtained by averaging a few support embeddings, which may lead to biased prototypes when support samples contain occlusion, motion blur, or incomplete action patterns. Such prototype shift becomes severe in sports images recognition, where fine-grained action categories exhibit strong visual overlap. To alleviate this limitation, Cross-Prototype Feature Refinement (CPFR) is proposed to calibrate prototypes and query embeddings through cross-prototype interactions, which improves the robustness of similarity measurement.

Figure 4 illustrates the CPFR module, whose computational procedure is described below: For an N-way K-shot episode, the support set is $D_{\sup}={\left\{(I_{n,k}^{\sup},y_{n}^{\sup})\right\}}_{n=1,k=1}^{N,K}$ , and the query set is $D_{\mathrm{que}}={\left\{I_{t}^{\mathrm{que}}\right\}}_{t=1}^{T}$ . Given refined support feature maps $X_{n,k}^{\sup}$ and a refined query feature map $X_{t}^{\mathrm{que}}$ , the detailed of CPFR are as follows:

First, a prototype feature map is obtained by averaging support features within each class, as defined in Equation 19:

$$F_{l}^{\left(n\right)}=\frac{1}{K}\sum_{k=1}^{K}X_{n,k}^{\sup}$$ (19)

Meanwhile, the query feature is aligned by a convolution layer, as shown in Equation 20:

$$F_{l-1}=\text{Conv}(X_{t}^{\mathrm{que}}),F_{l-1}\in R^{C\times H\times W}$$ (20)

Then, global descriptors are extracted by global average pooling, as defined in Equation 21:

$$g=\mathrm{GAP}(F_{l}^{\left(n\right)}),g\in R^{C}$$ (21)

Two fully connected layers are applied to generate two channel embeddings, as shown in Equation 22:

$$u={\mathrm{FC}}_{1}(g),v={\mathrm{FC}}_{2}(g),u,v\in R^{C}$$ (22)

After reshaping into C × 1 × 1, a cross-channel relation matrix is constructed by transpose multiplication followed by softmax normalization, as defined in Equations 23, 24:

$$U=\text{reshape}(u)\in R^{C\times 1},V=\text{reshape}(v)\in R^{C\times 1}$$ (23)

$$J=\text{Softmax}(U⊗V^{⊤}),R\in R^{C\times C}$$ (24)

Based on *J*, both the prototype and the query features are re-calibrated through channel-wise transformation, as defined in Equation 25:

$$\begin{array}{l} \hat{F_{l}^{\left(n\right)}}={\text{reshape}}^{-1}(J\;\text{reshape}(F_{l}^{\left(n\right)}));\\ \hat{F_{l-1}}={\text{reshape}}^{-1}(J^{⊤}\text{reshape}(F_{l-1})) \end{array}$$ (25)

Finally, feature aggregation is performed to obtain the refined prototype feature, as shown in Equation 26:

$$\bar{F_{l}^{\left(n\right)}}=\hat{F_{l}^{\left(n\right)}}+\hat{F_{l-1}},\bar{F_{l}^{\left(n\right)}}\in R^{C\times H\times W}$$ (26)

It should be noted that the refined prototype embedding and query embedding are computed as defined in Equation 27:

$$s_{t,n}=\mathrm{GAP}(\bar{F_{l}^{\left(n\right)}}),s_{t,n}\in R^{C};q_{t}=\mathrm{GAP}(X_{t}^{\mathrm{que}})$$ (27)

which are used for cosine similarity matching to predict the query label.

### 2.5 Loss function

To jointly enhance discriminative representation learning and prototype matching during meta-training, a hybrid objective is adopted, which combines a supervised classification loss and an episodic metric-based loss. Both losses are optimized only using training episodes sampled from 𝐷base, while 𝐷novel is reserved strictly for meta-testing. To be specific, for each training sample are produced by the classification head is defined in Equation 28:

$$L_{c\ell s}=-\frac{1}{|D_{\text{base}}|}\sum_{(I_{j},y_{j})\in D_{\text{base}}}\log\frac{\exp(o_{j,y_{j}})}{\sum_{c=1}^{C_{\text{base}}}\exp(o_{j,c})}$$ (28)

For each N-way K-shot episode, the support set $D_{\sup}={\left\{(I_{n,k}^{\sup},y_{n}^{\sup})\right\}}_{n=1,k=1}^{N,K}$ and query set $D_{\mathrm{que}}={\left\{I_{t}^{\mathrm{que}}\right\}}_{t=1}^{T}$ are constructed. Based on refined embeddings $q_{t}$ and $s_{t,n}$ , cosine scores are computed as defined in Equation 29:

$$\text{score}(q_{t},n)=\cos(q_{t},s_{t,n})$$ (29)

Then, the class probability of $I_{t}^{\mathrm{que}}$ is defined in Equation 30:

$$p(y=nI_{t}^{\mathrm{que}})=\frac{\exp(\text{score}(q_{t},n)/\tau )}{\sum_{j=1}^{N}\exp(\text{score}(q_{t},j)/\tau )}$$ (30)

where 𝜏 is a temperature coefficient. The metric-based loss is formulated as shown in Equation 31:

$$L_{m}=-\frac{1}{T}\sum_{t=1}^{T}\log p(y=y_{t}^{\mathrm{que}}I_{t}^{\mathrm{que}})$$ (31)

where $y_{t}^{\mathrm{que}}$ denotes the ground-truth label of the query image $I_{t}^{\mathrm{que}}$ . The overall learning objective is formulated as shown in Equation 32:

$$L=L_{c\ell s}+\lambda L_{m}$$ (32)

where *λ* is a weighting coefficient.

## 3 Experiments

### 3.1 Implementation details

DSCPNet is implemented in Python 3.8.13 with PyTorch 1.10.0. All experiments are conducted on a workstation equipped with an Intel Xeon Silver 4210R CPU and an NVIDIA RTX 3090 GPU with 24 GB memory. The model is optimized using Adam during training, with the weight decay configured as 0.001 and the momentum parameter set to 0.99. During meta-training, only base classes were used to optimize the model parameters. Since no separate validation classes were defined in the constrained sports-domain setting, hyperparameters were selected using validation episodes sampled only from the base-class data. The final novel classes were kept strictly unseen before meta-testing and were not used for model training or hyperparameter tuning. During meta-testing, K labeled images per novel class were randomly sampled as the support set, and 15 images per class were sampled as the query set. For each 5-way K-shot setting, 600 episodes were randomly sampled for evaluation.

### 3.2 Datasets

To demonstrate the effectiveness of DSCPNet in sports image recognition, two typical publicly available datasets, i.e., Sports 22 (Mizher et al., 2025) and Sports 100 (Li Q. et al., 2024), are adopted for evaluation. The specific statistics of the two datasets are shown in Table 1 and some examples of are displayed in Figure 5. For both datasets, the base classes were used for meta-training, while the novel classes were used only for final meta-testing. The support and query sets were constructed from the novel classes only during evaluation, and no novel-class images were used for hyper-parameter tuning.

1. The Sports22 dataset is a publicly available sports image classification benchmark collected from Internet sources and released on Kaggle, which contains images from 22 sports categories. In this study, 10 categories are selected to construct a constrained sports-domain few-shot evaluation setting, including baseball, basketball, boxing, cricket, football, gymnastics, swimming, tennis, volleyball, and weightlifting. All images are resized to 224 × 224 × 3. Among them, baseball, basketball, boxing, cricket, and football are used as the base classes for model training, while gymnastics, swimming, tennis, volleyball, and weightlifting are used as the novel classes for few-shot evaluation. The five novel classes were selected to construct a standard 5-way K-shot evaluation setting and to provide a controlled sports-domain test scenario. These novel classes cover representative sports visual patterns, including body-centered actions, ball-related cues, water or stadium backgrounds, and visually similar action/posture patterns, which are relevant to the scale variation, background distraction, and fine-grained inter-class ambiguity considered in this study.
2. Sports100 is a Kaggle sports image benchmark built from web-collected images and organized for large-category sports classification. It covers 100 human sports action categories, where duplicate images are filtered by a duplicate-detection algorithm to avoid data leakage across the training, validation, and test splits. All images are resized to 224 × 224 × 3. The dataset provides 13,493 training images and two balanced evaluation splits, including 500 validation images and 500 test images. In this study, ten categories are selected and further divided into five base classes and five novel classes to construct a constrained sports-domain few-shot evaluation setting. Specifically, baseball, basketball, boxing, cricket, and bowling are used as the base classes for training, while air hockey, archery, wrestling, bmx, and bobsled are used as the novel classes for few-shot evaluation. The selected novel classes include sports with diverse visual characteristics, such as object-centered scenes, distinctive body postures, background-dependent contexts, and visually similar action patterns. This selection is used to evaluate DSCPNet under the key challenges of scarce supervision, scale diversity, background distraction, and fine-grained inter-class ambiguity.

**Table 1:** Detailed information of the two sports datasets in this study.

Sports 22			Sports 100		
Type of sports	Number	Percent	Type of sports	Number	Percent
Total	5,894	100%	Total	2039	100%
Baseball	734	12.45%	Baseball	274	13.44%
Basketball	456	7.74%	Basketball	269	13.19%
Boxing	665	11.28%	Boxing	216	10.60%
Cricket	666	11.30%	Cricket	229	11.23%
Football	761	12.91%	Bowling	220	10.79%
Gymnastics	681	11.55%	Air hockey	212	10.40%
Swimming	651	11.04%	Archery	232	11.38%
Tennis	677	11.49%	Wrestling	149	7.31%
Volleyball	670	11.37%	Bmx	240	11.77%
Weightlifting	533	9.04%	Bobsled	198	9.71%

**Figure 5:**
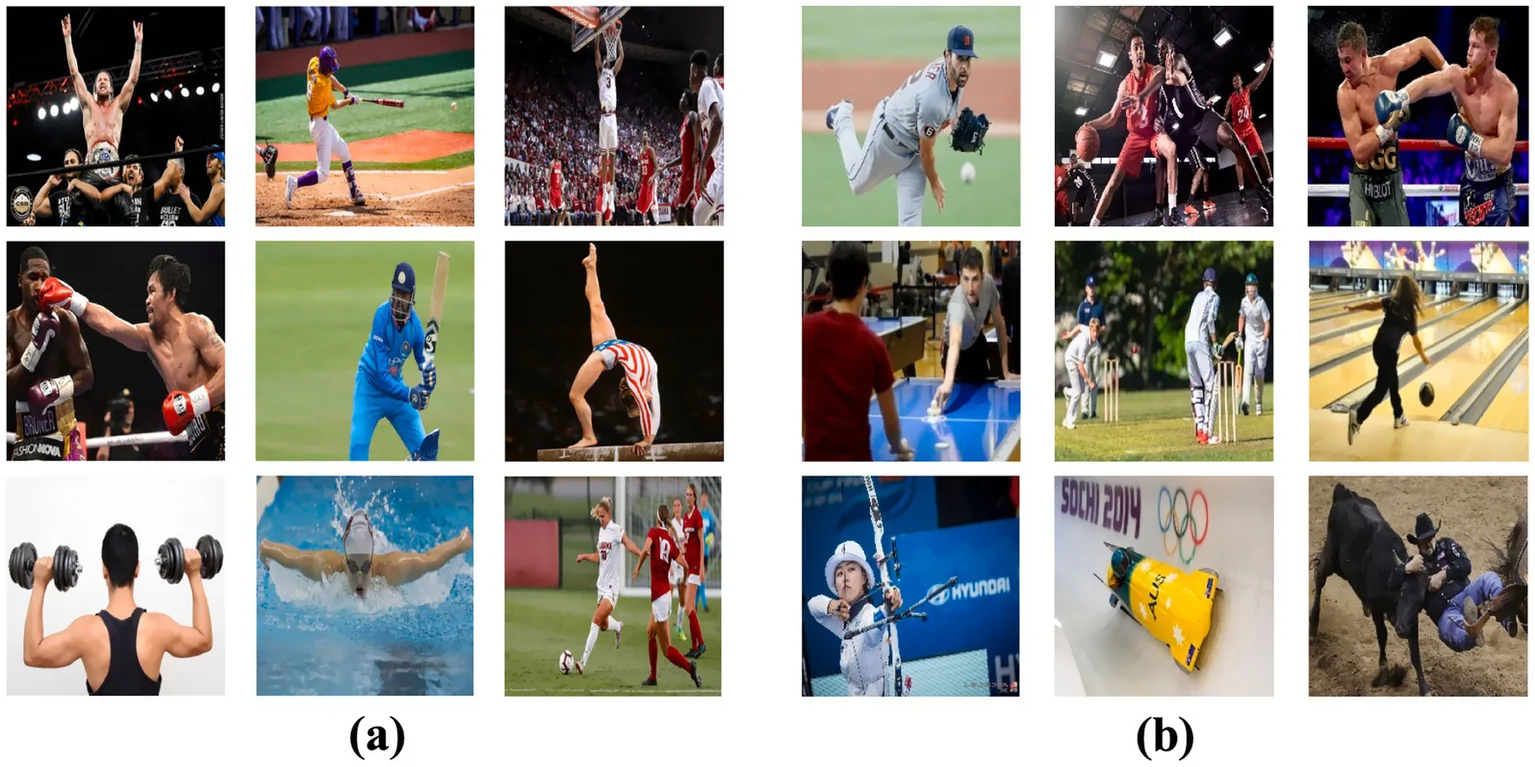
Examples from the two datasets. **(a)** Sports 22. **(b)** Sports 100.

It should be noted that the selected categories form a constrained sports-domain few-shot evaluation setting rather than a large-scale meta-learning benchmark with many base and novel classes. This setting was adopted to evaluate whether the proposed method can distinguish representative sports categories under scarce supervision. In this setting, the five novel classes are fixed during meta-testing, while support and query samples are randomly sampled across repeated test episodes. Therefore, although the novel class set remains fixed, the support-query combinations vary across episodes.

### 3.3 Assessment metrics

Classification accuracy (Acc) is adopted as the primary evaluation metric for comparing DSCPNet with the baseline models, and it is calculated as shown in Equation 33:

$$\mathrm{Acc}=\frac{\mathrm{TP}+\mathrm{TN}}{\mathrm{TN}+\mathrm{FP}+\mathrm{FN}+\mathrm{TP}}$$ (33)

where TP and TN represent correctly predicted positive and negative cases, whereas FP and FN indicate the corresponding misclassified cases. To reduce the influence of episode-level randomness, each 5-way K-shot evaluation was repeated over 600 randomly sampled test episodes. In each episode, K labeled samples per novel class were used as the support set, and 15 images per class were used as the query set. The final results are reported as mean accuracy with standard deviation and 95% confidence intervals. The 95% confidence interval is calculated as $1.96\times \frac{\sigma }{\sqrt{600}}$ , where σ denotes the standard deviation of accuracy across the 600 test episodes.

### 3.4 Experimental results and analysis

#### 3.4.1 Quantitative and visual results

To illustrate the effectiveness of DSCPNet, several representative few-shot classification methods, including DeepEMD (Zhang et al., 2020), FaNet (Zhao et al., 2023), FewVS (Li Z. et al., 2024), CPEA (Hao et al., 2023), HMRM (Jin et al., 2024), PRO-CLIP (Cao et al., 2024), IRMAE-AKDE (Zhang et al., 2025), and ProtoNet (Snell et al., 2017) are chosen for performance comparison on Sports22 and Sports100. In the original-backbone comparison, the backbone of each compared method follows the best-performing or officially recommended configuration reported in its original implementation. This setting was adopted to compare DSCPNet with representative methods under their commonly used and strong configurations. Since different backbone architectures may affect the interpretation of performance differences, a controlled comparison under the same ResNet12 backbone is further conducted with the same training and testing protocol. The original-backbone comparison results are reported in Table 2, while the same-backbone controlled comparison is reported in Table 3. As reported in Table 2, DSCPNet obtains 84.54% accuracy in the 5-way 5-shot task and 70.61% in the 5-way 1-shot task, improving over IRMAE-AKDE and PRO-CLIP by 2.71 and 2.26%, respectively. Table 3 further demonstrates that DSCPNet consistently attains the highest accuracy on Sports100, reaching 89.47% (5-shot) and 76.83% (1-shot), which are 3.64 and 3.68% higher than IRMAE-AKDE, respectively. These gains on both datasets and under different shot settings indicate that DSCPNet provides a robust solution for few-shot sports image recognition. All methods in Table 3 are evaluated under the same ResNet12 backbone and the same training and testing protocol. ProtoNet is included as a standard prototype-based few-shot baseline, which provides a direct reference for evaluating whether the proposed cascaded prototype refinement strategy improves conventional prototype matching. As shown in Table 3, DSCPNet achieves the best performance under all four evaluation settings. Specifically, DSCPNet obtains 84.54 and 70.61% on Sports22 under the 5-way 5-shot and 5-way 1-shot settings, respectively, and 89.47 and 76.83% on Sports100. Compared with the strongest baseline IRMAE-AKDE, DSCPNet improves the accuracy by 1.08%, 1.24%, 1.66%, and 1.61% under the four settings, respectively. These results indicate that the performance improvement is not solely caused by backbone differences, but is also related to the proposed dynamic scale fusion and cascaded prototype refinement strategy. To further evaluate the statistical robustness of DSCPNet under the constrained five-way evaluation setting, the confidence interval results are summarized in Table 4. As shown in Table 4, DSCPNet obtains narrow 95% confidence intervals across the repeated test episodes on both datasets. This indicates that the reported performance is relatively stable with respect to support-query sampling randomness under the evaluated five-way settings.

**Table 2:** Classification accuracy on Sports 22 and Sports100 dataset compared with other methods.

Method	Backbone	Sports 22		Sports 100	
		5-way 5-shot	5-way 1-shot	5-way 5-shot	5-way 1-shot
ProtoNet	ResNet12	76.82 ± 0.19	64.08 ± 0.44	78.91 ± 0.21	66.74 ± 0.26
DeepEMD	ResNet12	77.16 ± 0.12	64.51 ± 0.52	79.46 ± 0.18	67.21 ± 0.22
FaNet	ResNet18	78.32 ± 0.22	65.18 ± 0.15	80.93 ± 0.22	68.98 ± 0.34
FewVS	ViT-S	79.46 ± 0.37	66.24 ± 0.13	82.54 ± 0.45	70.74 ± 0.13
CPEA	ViT-S	80.21 ± 0.42	66.79 ± 0.11	83.15 ± 0.17	71.45 ± 0.24
HMRM	ResNet12	80.63 ± 0.34	67.43 ± 0.25	84.21 ± 0.15	72.14 ± 0.15
PRO-CLIP	ViT-B	81.08 ± 0.11	67.94 ± 0.12	84.94 ± 0.15	72.79 ± 0.42
IRMAE-AKDE	ResNet18	81.83 ± 0.26	68.35 ± 0.29	85.83 ± 0.22	73.15 ± 0.25
DSCPNet	ResNet12	84.54 ± 0.37	70.61 ± 0.27	89.47 ± 0.14	76.83 ± 0.29

**Table 3:** Controlled comparison under the same ResNet12 backbone.

Method	Backbone	Sports22		Sports100	
		5-way 5-shot	5-way 1-shot	5-way 5-shot	5-way 1-shot
ProtoNet	ResNet12	76.82 ± 0.19	64.08 ± 0.44	78.91 ± 0.21	66.74 ± 0.26
DeepEMD	ResNet12	77.16 ± 0.12	64.51 ± 0.52	79.46 ± 0.18	67.21 ± 0.22
FaNet	ResNet12	78.35 ± 0.31	65.09 ± 0.36	80.72 ± 0.16	68.06 ± 0.19
FewVS	ResNet12	79.08 ± 0.24	65.88 ± 0.41	82.15 ± 0.20	69.43 ± 0.24
CPEA	ResNet12	80.21 ± 0.29	66.96 ± 0.33	83.37 ± 0.17	71.36 ± 0.18
HMRM	ResNet12	80.63 ± 0.34	67.43 ± 0.25	84.21 ± 0.15	72.14 ± 0.15
PRO-CLIP	ResNet12	82.07 ± 0.28	68.14 ± 0.30	85.62 ± 0.19	73.31 ± 0.21
IRMAE-AKDE	ResNet12	83.46 ± 0.35	69.37 ± 0.23	87.81 ± 0.13	75.22 ± 0.27
DSCPNet	ResNet12	84.54 ± 0.37	70.61 ± 0.27	89.47 ± 0.14	76.83 ± 0.29

**Table 4:** Statistical robustness of DSCPNet over 600 test episodes.

Dataset	Setting	Accuracy	Std	95% CI
Sports22	5-way 5-shot	84.54	0.37	±0.03
	5-way 1-shot	70.61	0.27	±0.02
Sports100	5-way 5-shot	89.47	0.14	±0.01
	5-way 1-shot	76.83	0.29	±0.02

#### 3.4.2 Visualization comparison

To further demonstrate the superiority of DSCPNet, t-SNE and normalized confusion matrices are utilized for qualitative visualization on Sports22 and Sports100. Specifically, the t-SNE results in Figures 6 and 7 show that DSCPNet achieves more compact intra-class clusters and clearer inter-class boundaries than competing few-shot methods, while the baseline features are relatively scattered with noticeable overlaps. In addition, the normalized confusion matrices in Figures 8 and 9 further verify the class-wise recognition performance of DSCPNet under 5-shot and 1-shot settings. As shown in Figure 8, the diagonal entries increase by 13%, 16%, 14%, 16%, and 12% for Gymnastics, Swimming, Tennis, Volleyball, and Weightlifting, respectively, when switching from 1-shot to 5-shot. Meanwhile, several representative misclassification terms are markedly reduced, including decreases of 3% for Gymnastics being predicted as Swimming, 4% for Gymnastics being predicted as Volleyball, 4% for Swimming being predicted as Gymnastics, and 5% for Volleyball being predicted as Tennis. For Sports100 in Figure 9, the diagonal responses rise by 12%, 14%, 12%, 15%, and 10% for Air hockey, Archery, Wrestling, Bmx, and Bobsled, respectively, accompanied by reduced off-diagonal errors, such as decreases of 3% for Air hockey being predicted as Bmx, 4% for Archery being predicted as Air hockey, and 4% for Bmx being predicted as Wrestling. Overall, these visualizations indicate that DSCPNet learns more discriminative representations and alleviates inter-class ambiguity on both datasets, particularly when more labeled support samples are available.

**Figure 6:**
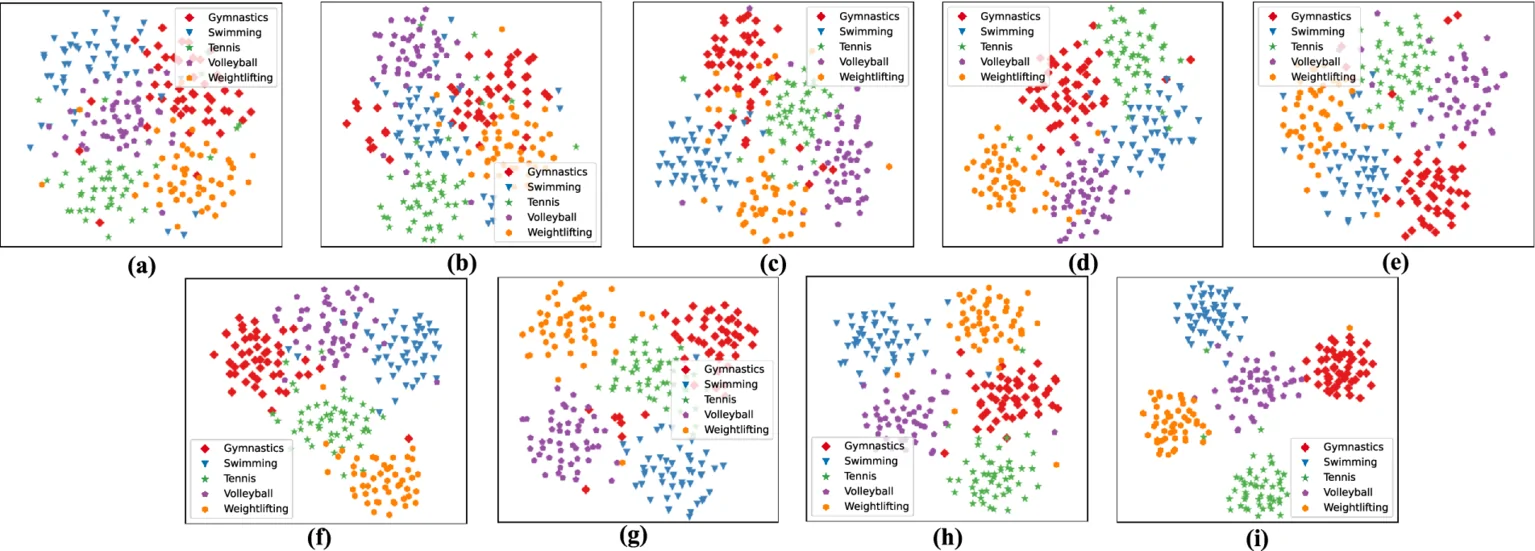
t-SNE visualization of DSCPNet and other FSL methods on Sports 22. **(a)** ProtoNet. **(b)** DeepEMD. **(c)** FaNet. **(d)** FewVS. **(e)** PRO-CLIP. **(f)** CPEA. **(g)** HMRM. **(h)** IRMAE-AKDE. **(i)** DSCPNet.

**Figure 7:**
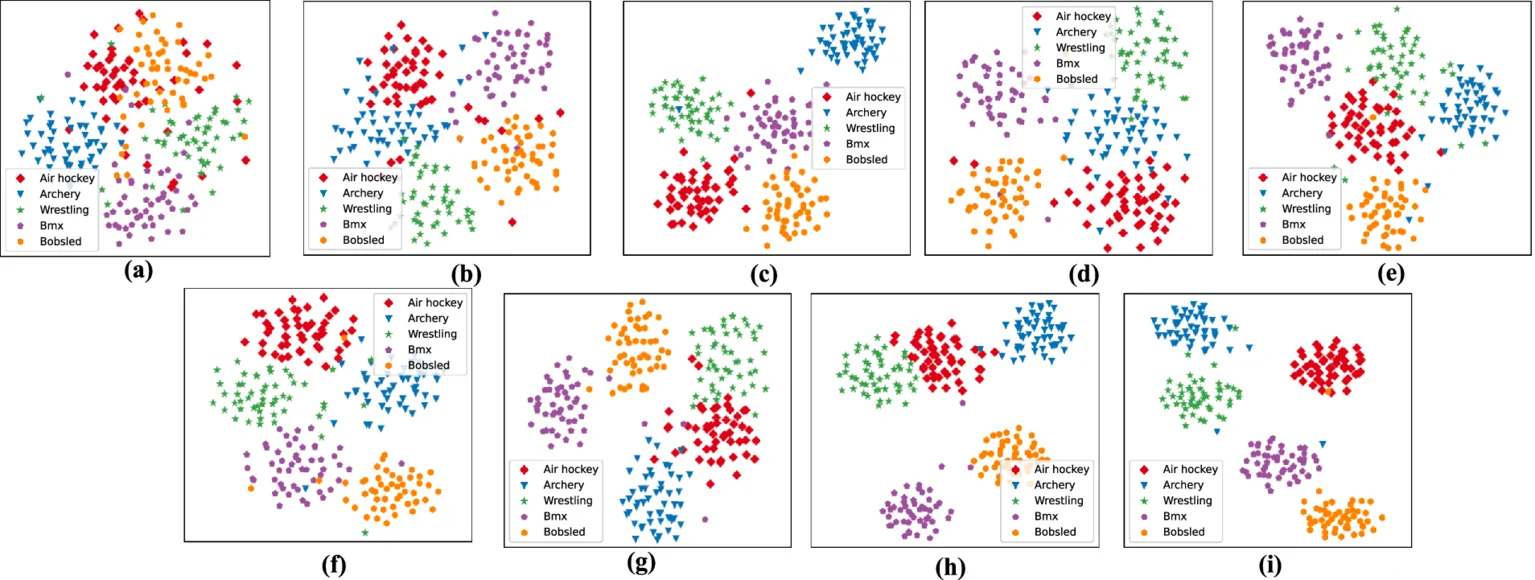
t-SNE visualization of DSCPNet and other FSL methods on Sports 100. **(a)** ProtoNet. **(b)** DeepEMD. **(c)** FaNet. **(d)** FewVS. **(e)** PRO-CLIP. **(f)** CPEA. **(g)** HMRM. **(h)** IRMAE-AKDE. **(i)** DSCPNet.

**Figure 8:**
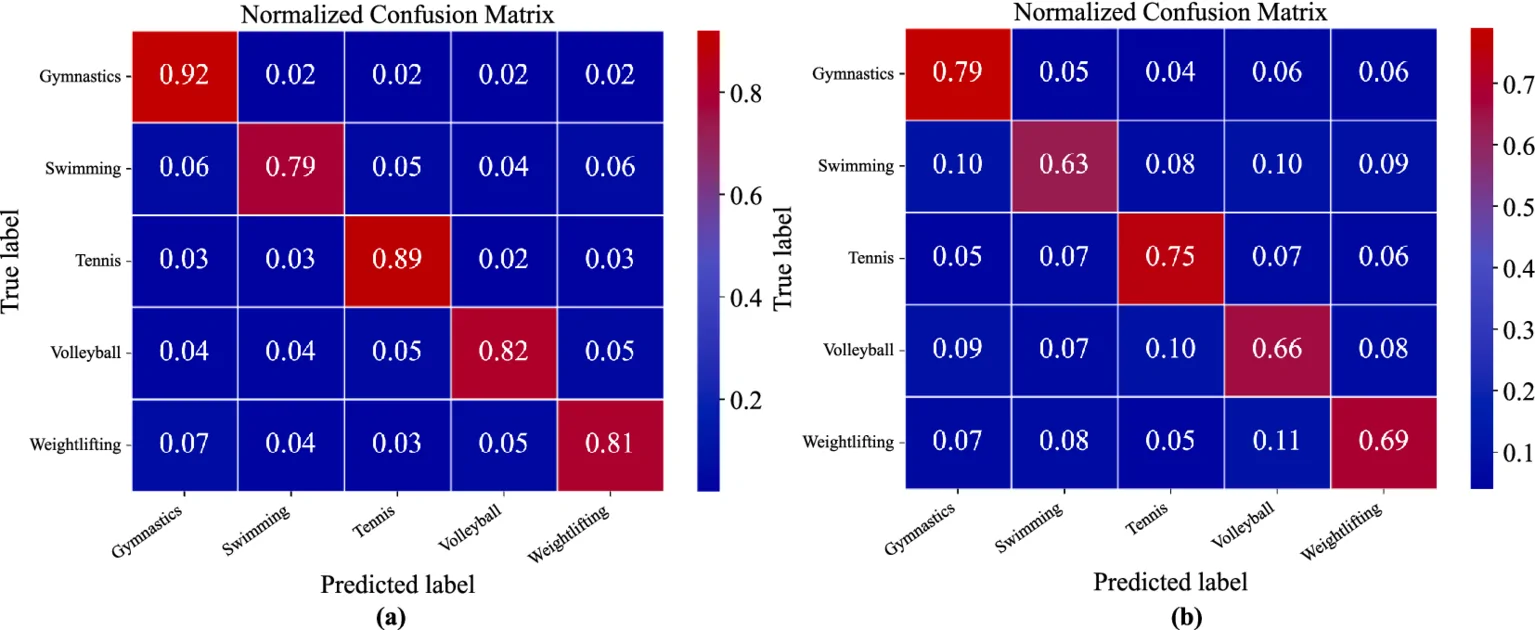
Confusion matrix of DSCPNet on Sports22. **(a)** 5-shot. **(b)** 1-shot.

**Figure 9:**
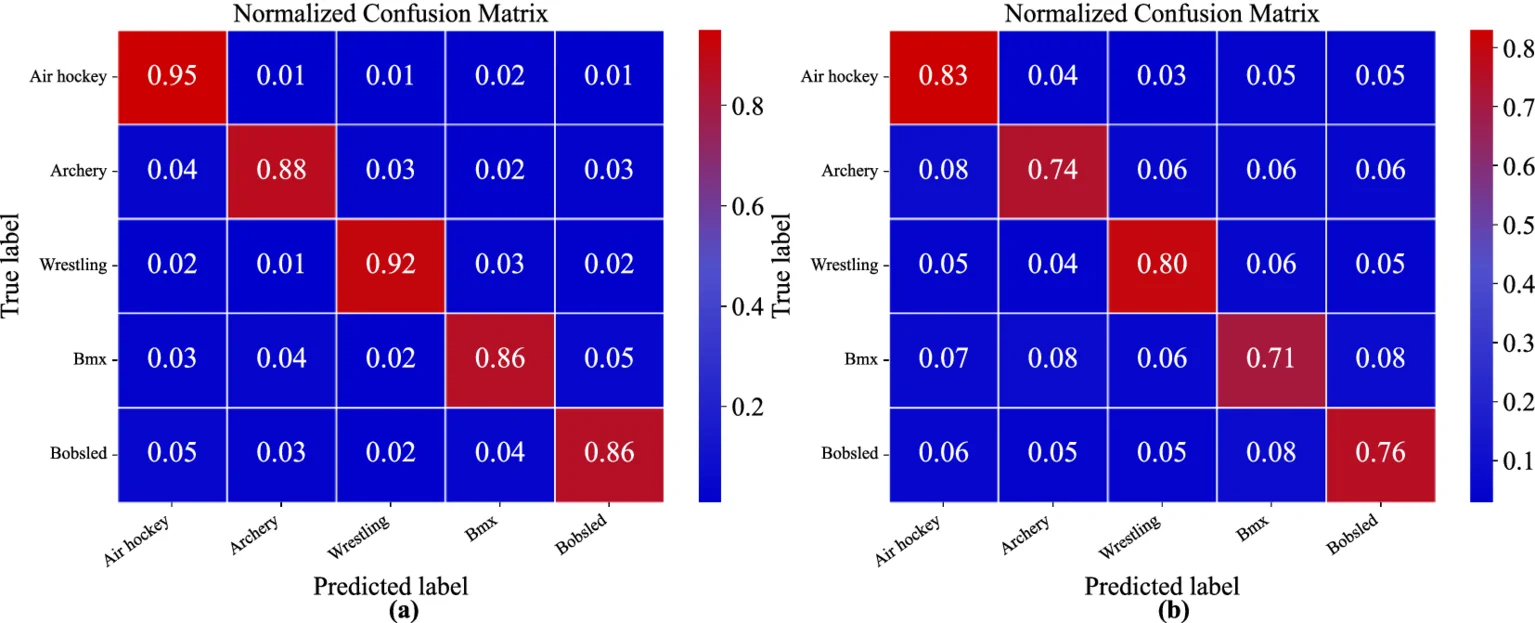
Confusion matrix of DSCPNet on Sports 100. **(a)** 5-shot. **(b)** 1-shot.

#### 3.4.3 Analysis of computational complexity

To analyze the computational complexity of different few-shot sports image classification methods, three commonly used metrics, i.e., the number of parameters (Params), floating-point operations (FLOPs), and memory consumption (Memory), are adopted, and the results are summarized in Table 4. It can be observed that lightweight baselines such as FaNet require the fewest parameters and the lowest FLOPs, while large-scale models such as PRO-CLIP and IRMAE-AKDE exhibit substantially higher computational cost and memory usage. Compared with existing methods, DSCPNet maintains a moderate model size with 89.44 MB parameters and 95.12 G FLOPs, and its memory footprint is 307.41 MB, indicating a balanced complexity profile. Overall, the results suggest that DSCPNet achieves a reasonable trade-off between computational overhead and resource consumption, which is suitable for practical few-shot sports image classification scenarios (see Table 5).

**Table 5:** Computational complexity comparison.

Method	Params (MB)	FLOPS (G)	Memory (MB)
ProtoNet	4.85	66.74	118.52
DeepEMD	6.36	50.31	226.13
FaNet	5.31	8.31	137.25
FewVS	12.19	74.21	249.74
PRO-CLIP	143.32	200.49	637.39
CPEA	44.39	64.73	225.29
HMRM	90.72	497.54	734.41
IRMAE-AKDE	150.54	215.43	624.57
DSCPNet	89.44	95.12	307.41

### 3.5 Ablative experiments

#### 3.5.1 Ablation study of DSFA, CFSI, and CPFR

Ablation experiments on Sports22 and Sports100 are performed to quantify the effect of each DSCPNet component, with the corresponding results summarized in Table 6, where DSFA, CFSI, and CPFR denote Dynamic Scale Fusion Attention, Cascaded Foreground-Scene Interaction, and Cross-Prototype Feature Refinement, respectively. In Table 6, M1 denotes the baseline model, which uses the same ResNet12 backbone and episodic training/testing protocol as DSCPNet but removes DSFA, CFSI, and CPFR. M2-M4 add DSFA, CFSI, and CPFR individually, respectively. M5-M7 evaluate different two-module combinations, and M8 denotes the complete DSCPNet.

**Table 6:** Results of ablation study.

Methods	DSFA	CFSI	CPFR	Sports 22		Sports 100	
				5-way 5-shot	5-way 1-shot	5-way 5-shot	5-way 1-shot
M1	—	—	—	72.47 ± 0.09	58.48 ± 0.16	78.13 ± 0.25	64.27 ± 0.15
M2	√	—	—	78.06 ± 0.18	63.82 ± 0.36	83.94 ± 0.14	69.74 ± 0.04
M3	—	√	—	76.25 ± 0.13	61.50 ± 0.26	81.05 ± 0.54	67.25 ± 0.32
M4	—	—	√	73.92 ± 0.23	60.03 ± 0.01	80.23 ± 0.35	66.04 ± 0.12
M5	√	√	—	82.78 ± 0.04	68.84 ± 0.21	87.05 ± 0.27	74.89 ± 0.29
M6	√	—	√	82.12 ± 0.33	68.11 ± 0.47	86.25 ± 0.31	74.15 ± 0.23
M7	—	√	√	81.49 ± 0.21	67.36 ± 0.13	84.74 ± 0.18	73.09 ± 0.31
M8	√	√	√	84.54 ± 0.37	70.61 ± 0.27	89.47 ± 0.14	76.83 ± 0.29

As shown in Table 6, each proposed module improves the baseline performance when used individually. Compared with M1, M2 improves the accuracy from 72.47 to 78.06% and from 58.48 to 63.82% on Sports22 under the 5-way 5-shot and 5-way 1-shot settings, respectively. On Sports100, M2 also improves the accuracy from 78.13 to 83.94% and from 64.27 to 69.74%. These results indicate that DSFA contributes substantially to feature representation under sports images with scale variation. M3 improves the baseline accuracy from 72.47 to 76.25% and from 58.48 to 61.50% on Sports22, and from 78.13 to 81.05% and from 64.27 to 67.25% on Sports100, suggesting that CFSI is beneficial for improving feature discrimination in sports scenes with background distraction. M4 also improves the baseline accuracy to 73.92%, 60.03%, 80.23%, and 66.04% under the four settings, indicating that CPFR helps improve prototype calibration and similarity estimation for fine-grained recognition.

The two-module variants M5-M7 further improve the performance compared with the corresponding single-module variants. Among them, M5 achieves stronger results than M2 and M3, M6 outperforms M2 and M4, and M7 improves over M3 and M4, demonstrating the complementary effects among DSFA, CFSI, and CPFR. Finally, M8 achieves the best performance under all four evaluation settings, reaching 84.54%, 70.61%, 89.47%, and 76.83%, respectively. These results indicate that the final performance gain of DSCPNet comes from the joint contribution of dynamic scale fusion, foreground-scene interaction, and cross-prototype feature refinement.

### 3.6 Sensitive analysis

#### 3.6.1 Influence of different shots

To investigate the influence of the number of shots, experiments with different shot settings are conducted, and the results on Sports22 and Sports100 are presented in Figure 10. As shown in Figure 10, the recognition accuracy consistently increases as more support samples are provided, which indicates that all methods can exploit additional supervision to learn more reliable class representations. Moreover, DSCPNet achieves the best performance across all shot numbers on both datasets, demonstrating its strong capability of extracting discriminative cues and maintaining stable generalization under varying degrees of data scarcity. In particular, the performance gap between DSCPNet and competing methods remains evident from low-shot to relatively higher-shot regimes, which validates the effectiveness of DSCPNet in few-shot sports image classification when the available labeled samples gradually increase.

**Figure 10:**
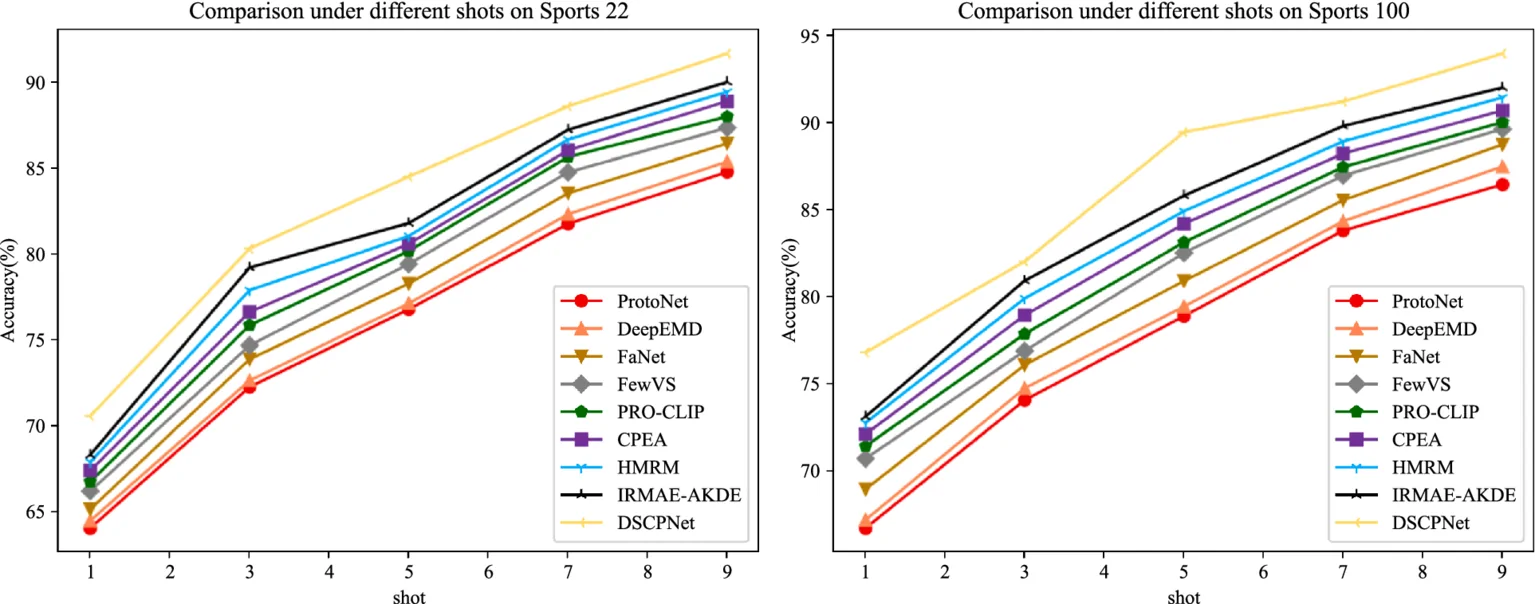
Experiment results under different shots.

#### 3.6.2 Influence of backbones

To investigate the impact of different backbones on DSCPNet, five representative architectures, i.e., ResNet12, ResNet18, WRN-28-10, ResNet50, and ResNet101, are adopted as the feature encoder and evaluated on Sports22 and Sports100 under 1-shot and 5-shot settings. Experiment outcomes are shown in Table 7. It is clear that WRN-28-10 achieves the best accuracy consistently across both datasets and both shot settings, indicating that a stronger backbone is beneficial for learning more discriminative representations in few-shot sports image classification. In contrast, ResNet50 and ResNet101 lead to noticeable performance drops, suggesting that larger-capacity backbones may not always improve generalization under limited support samples. Moreover, the inference time increases with the model capacity. Although ResNet18 and WRN-28-10 provide higher accuracy than ResNet12, they also introduce higher computational cost. Therefore, considering the trade-off between recognition performance and efficiency, ResNet12 is selected as the default backbone of DSCPNet in the remaining experiments.

**Table 7:** Comparative experiments of different backbones.

Backbone	Sports 22		Sports 100	
	5-way 5-shot	5-way 1-shot	5-way 5-shot	5-way 1-shot
ResNet12	84.54 ± 0.37/3.57	70.61 ± 0.27/1.53	89.47 ± 0.14/3.13	76.83 ± 0.29/1.45
ResNet18	85.43 ± 0.14/5.32	73.17 ± 0.22/2.85	92.14 ± 0.31/4.32	78.41 ± 0.24/2.14
WRN-28-10	86.15 ± 0.23/8.53	74.39 ± 0.17/5.36	93.35 ± 0.03/7.43	79.79 ± 0.19/4.28
ResNet50	82.67 ± 0.27/9.46	68.32 ± 0.14/6.82	87.43 ± 0.01/8.35	75.42 ± 0.13/5.67
ResNet101	81.81 ± 0.14/12.29	67.14 ± 0.19/10.45	86.25 ± 0.09/11.56	74.21 ± 0.20/9.31

## 4 Limitations and discussion

Although DSCPNet achieves competitive performance in the evaluated settings, several limitations remain. First, the few-shot evaluation is conducted on selected categories from Sports22 and Sports100. The fixed five-way novel-class setting provides a controlled sports-domain evaluation scenario, but it reduces the diversity of class combinations compared with conventional few-shot benchmarks that contain many base and novel classes. Although 600 repeated test episodes are used to reduce support-query sampling randomness, broader class partitions and more diverse episodic evaluations should be investigated in future work. Second, the two datasets are web-collected sports image benchmarks, and therefore more real-world sports scenarios should be considered to further evaluate the robustness of the method. Third, DSCPNet introduces additional computational operations through DSFA, CFSI, and CPFR. Although ResNet12 is adopted as the default backbone to balance accuracy and efficiency, more lightweight designs should be explored for practical deployment. Finally, the current evaluation focuses on sports-domain few-shot classification. Extending DSCPNet to cross-domain sports scene recognition, fine-grained event recognition, and other sports understanding tasks will be considered in future work.

## 5 Conclusion

In this article, a novel few-shot sports image recognition framework, DSCPNet, is developed to address the challenges caused by limited labeled samples, scale variation, background distraction, and fine-grained inter-class ambiguity in sports images. First, Dynamic Scale Fusion Attention (DSFA) is developed to dynamically select and fuse contextual cues under different receptive fields, which enhances the scale-adaptive representation ability of the model under scarce supervision. Second, Cascaded Foreground–Scene Interaction (CFSI) is introduced to suppress irrelevant background interference through cascaded interaction modulation, thereby strengthening target-focused embeddings and reducing intra-class variance. Third, Cross-Prototype Feature Refinement (CPFR) is designed to jointly calibrate support prototypes and query representations in a cross-prototype manner, which produces more discriminative class prototypes and improves the robustness of similarity estimation for fine-grained recognition. Extensive experiments on two few-shot sports image classification benchmarks show the effectiveness of DSCPNet. The results further show that DSCPNet delivers higher accuracy than recent comparison methods in both low-shot evaluation settings, indicating its competitive performance in handling complex sports scenes with limited annotations. These characteristics also make the proposed framework potentially useful for sport psychology-related applications, such as athlete behavior analysis, training feedback, exercise participation monitoring, and data-driven sport behavior studies under limited annotation conditions. Future work will focus on developing a more lightweight DSCPNet, improving robustness under more realistic sports data conditions, and extending the framework to broader few-shot sports understanding tasks such as fine-grained event recognition and cross-domain sports scene classification.

## Data Availability

The original contributions presented in the study are publicly available. All the data used in the article can be found in the following links: Sports 22: https://www.kaggle.com/datasets/sheikhzaib/sports-image-image-classification, and Sports 100: https://www.kaggle.com/datasets/gpiosenka/sports-classification.
